# Internet addiction and suicidal behavior among vocational high school students in Hunan Province, China: A moderated mediation model

**DOI:** 10.3389/fpubh.2022.1063605

**Published:** 2023-01-10

**Authors:** Ziwei Teng, Yaru Zhang, Zirou Wei, Mengdong Liu, Meidai Tang, Yizhi Deng, Zhuohui Chen, Ying Wu, Runqi Liu, Yuanguang Yang, Menghui Gao, Jin Kun, Jindong Chen, Renrong Wu, Jing Huang

**Affiliations:** ^1^Department of Psychiatry, National Clinical Research Center for Mental Disorders, The Second Xiangya Hospital of Central South University, Changsha, Hunan, China; ^2^Mental Health Center, The Second Affiliated Hospital of Guangxi Medical University, Nanning, China; ^3^Department of Psychology, University of Washington, Seattle, WA, United States; ^4^Department of Psychiatry, Chenzhou No. 2 Middle School, Chenzhou, Hunan, China; ^5^Department of Neurosurgery, Xiangya Hospital, Central South University, Changsha, Hunan, China; ^6^Department of Intensive Care Unit, The Second Xiangya Hospital, Central South University, Changsha, Hunan, China; ^7^Department of Psychiatry, The Third Peoples Hospital of Tongren, Tongren, China

**Keywords:** anxiety, depression, Internet addiction, stress, suicidal behavior, vocational education

## Abstract

**Background:**

Vocational education is an important part of high school education in China. However, there is little research on high school students' mental health. This study aimed to investigate the prevalence of suicidal behavior (SB) among this population and the mediating role of insomnia, depression, anxiety, and stress in the relationship between Internet addiction (IA) and SB using a structural equation model.

**Methods:**

A cross-sectional questionnaire survey was conducted among several vocational high school students in Hunan Province, and 7,968 valid questionnaires were obtained. General demographic data and data from the Dual-Mode Self-Control Scale, Athens Insomnia Scale, Depression Anxiety Stress scale-21, and Revised Chen Internet Addiction Scale were collected. A structural equation model was used to explore the different pathways from IA to SB.

**Results:**

Among the participants, 37.7, 15.7, and 21.8% reported suicidal ideation, plans, and attempts, respectively. The structural equation model confirmed that IA was indirectly related to SB and was mediated by insomnia and/or depression, anxiety, and stress.

**Limitations:**

First, we only recruited students from vocational schools in Hunan Province, therefore, the sample may not represent the entire population of vocational students in China. Second, self-report scales were used in this study, and clinical diagnosis required professional interviews. Third, since this study had a cross-sectional design, the causal relationship between the variables could not be determined.

**Conclusions:**

The prevalence of SB among vocational high school students in China was significantly high. The prevention of SB related to IA can be attributed to the improvement of insomnia and emotional problems.

## 1. Introduction

Suicide is a leading cause of death worldwide (1, 2). Suicidal behaviors (SB), such as suicidal ideation, plans, or attempts, are associated with various disabilities and social function impairment (3, 4). Adolescents are at an important stage of psychological development and exhibit high rates of suicide during this phase (3). From a multilevel approach, SB among adolescents is associated with different sociocultural, psychopathological, physiological, and biological factors (5). Evidence shows that depression (6), anxiety, and stress (DAS) are important risk factors for SB. More than 50% of the people who die by suicide have major depression (7), and almost all anxiety disorders are associated with increased suicide risk (8). Insomnia is the most common sleep disorder among adolescents (9), defined as a difficulty in initiating or maintaining sleep or waking up unusually early in the morning, resulting in an inability to get a satisfactory amount and/or quality of sleep (10). Insomnia seriously affects adolescents' physical and mental health and is a risk factor for the initiation and maintenance of various emotional problems, specifically anxiety and depression (11). Insomnia is also associated with increased suicide risk (12).

The number of Internet users has increased substantially over the last decade. According to a report from the China Internet Network Information Center, it reached 1.011 billion in 2021, with 183 million underage users (13). Internet addiction (IA) is a concerning phenomenon. IA (also referred to as “pathological Internet use,” “excessive Internet use,” “Internet dependence,” and “compulsive Internet use”) is characterized by excessive or poorly controlled urges or behaviors regarding Internet access (14). Recent research suggests that individuals with higher levels of impulsivity may show more Internet use (15). Therefore, we hypothesized that high impulsivity is related to IA. Previous studies showed significant associations between IA and psychological disorders such as stress, depression, anxiety, insomnia, physical illness, loneliness, and suicidal attempts (16–20). Previous studies have reported some association between IA and SB, and these studies suggested that participants with IA generally have a higher rate of SB (21–24). However, the mechanism underlying this association remains unclear.

Vocational education is an important component of high school education in China. Students receive a 3-year vocational/technical curriculum after graduating from junior high school and account for ~50% of the high school population. A few studies have found that compared with ordinary high school students, vocational school students are more prone to anxiety, impulsive tendency, physical symptoms (25), and have more self-injurious behaviors (26) and a higher probability of suicidal ideation (27). However, very few studies have examined the SB of vocational school students in China. Moreover, Chinese vocational high schools do not strictly control students' use of mobile phones and electronic devices, and hence, they may have easy access to the Internet. However, currently, studies on IA and related psychological problems in this population are insufficient.

Given that the strong association between insomnia and DAS is associated with IA and SB, they may be key mediators of the relationship between IA and SB. Although there is a bidirectional causal relationship between insomnia and DAS, we speculate that students with IA experience insomnia before DAS, which increases the risk of SB. More detailed theoretical models are needed to explain how IA affects SB, which is of great interest to the authors. Therefore, this study aimed to explore the relationship between IA, insomnia, DAS, and SB among Chinese vocational high school students. This study aimed to (1) investigate the prevalence of SB among Chinese vocational high school students, (2) explore the relationship between impulsivity and IA, and (3) use a structural equation model (SEM) to explore the mediating role of insomnia and DAS in the relationship between IA and SB.

## 2. Materials and methods

### 2.1. Participants

We employed a cross-sectional design. Three vocational high schools in Hunan, China were selected by convenience sampling method. A printed version of the questionnaire was distributed by college teachers to all the students. Of the 8,021 students, 34 refused to participate. A total of 7,987 vocational high school students were screened, of which 19 were excluded because they failed the “trick” question. Finally, 7,968 valid questionnaires were included. All students were informed of their participation and that they could withdraw at any time. All the materials were anonymized to protect the participants' privacy. Before providing instructions to students, these college counselors received training on how to guide students in filling out questionnaires.

### 2.2. Measures

#### 2.2.1. Basic sociodemographic characteristics

In this study, we used a self-designed questionnaire to collect sociodemographic variables, including age, sex, residence (urban/rural), and annual family income (< 100,000 yuan/more than 100,000 yuan).

#### 2.2.2. Athens insomnia scale

Symptoms of insomnia were assessed using the AIS, which contains eight self-reported items (28). Higher scores indicate a higher risk of insomnia. Cronbach's alpha for the Chinese version of the AIS was 0.912 (29).

#### 2.2.3. Depression, anxiety, and stress scale-21

The Chinese version of the DASS-21 was used, which includes the three dimensions of anxiety, depression, and stress (30). Each dimension was composed of seven items, and each item was scored from 0 to 3 points (“0” means it has not occurred in a week; “1” means 1 or 2 times a week; “2” means 3 or 4 times a week; “3” means≥5 times a week). The Chinese version of the DASS-21 has good reliability and validity among Chinese students (30).

#### 2.2.4. Dual-modes of self-control scale

Impulsivity levels were assessed using the DMSC-S, a scale comprising 12 items that assessed impulsivity levels (31). Each item was rated from 1 (not at all true) to 5 (very true). The higher the score, the higher the level of the individual's impulse system. Cronbach's coefficient for this scale was 0.82.

#### 2.2.5. The Revised chen Internet addiction scale

IA was assessed using the CIAS-R, which contains 19 self-report items. Each item is rated on a 4-point Likert scale (complete in conformity = 1, comparatively in conformity = 2, comparatively conformity = 3, and complete conformity = 4). The total score on the scale ranges from 0 to 76. Participants were considered to have IA if their scores reached or exceeded 53. The CIAS-R includes the IA core symptom (IA-Sym) and related problems (IA-RP) subscales. Among them, IA-Sym was divided into two factors: compulsive Internet use and IA withdrawal response (Sym-C and Sym-W) and IA tolerance (Sym-T), and IA-RP was divided into interpersonal and health problems (RP-IH) and time management problems (RP-TM). The CIAS-R has been reported to have good reliability and validity among Chinese adolescents (32).

#### 2.2.6. Suicidal behavior

SB includes suicidal ideation, plans, and attempts. In this study, they were measured using the following questions: “Have you ever had thoughts of committing suicide?”, “Have you ever made a suicide plan?”, and “Have you ever tried to die by suicide?” If the answer to the suicide attempt questions was yes, further questions were asked about the frequency and details. These questions are commonly used in suicide research worldwide (33).

### 2.3. Ethics statement and data collection

This study was approved by the ethics committee of Second Xiangya Hospital, Central South University, China. All participants who volunteered to participate were informed of the purpose, process, benefits, and risks.

### 2.4. Statistical analysis

Frequencies and proportions for categorical variables or means (SD) for continuous variables were used to describe participant characteristics. χ2 tests or 2-tailed independent sample *t*-tests were used to compare the distribution between male and female participants according to different characteristics. Spearman's correlation analysis was performed to examine the association between the psychological variables (impulsivity, insomnia, DAS, and IA) and SB. Finally, the serial multiple mediation hypothesis for impulsivity, insomnia, DAS, IA, and SB was tested, and path models utilizing the maximum likelihood (ML) approach were performed. Considering that the adopted scale scores were continuous variables, and the measures of SB were dichotomized, standardized coefficients, total effects, and indirect effects were reported. Model fit indices [root mean square error of approximation (RMSEA), comparative fit index (CFI), Tucker–Lewis index (TLI), and Standardized Root Mean Square Residual (SRMR)] were calculated to assess the model's goodness of fit. RMSEA (34) and SRMR (35) values < 0.08, CFI (36), and TLI (37) values > 0.90, indicated acceptable goodness of fit.

We used IBM SPSS Statistics 24.0 (IBM Corp., Armonk, NY, USA) to conduct descriptive and pairwise correlation analyses. Mplus Version 8.3 was used to conduct the path analysis. *P* < 0.05 were considered statistically significant.

## 3. Results

### 3.1. Sample characteristics

The sample characteristics are presented in Tables 1–3. Among 7,968 eligible participants, 3,002 (37.7%), 1,247 (15.7%), and 1,739 (21.8%) reported having suicidal ideation, plans, and attempts, respectively. Participants with suicidal ideation/attempts were younger than those without (*P* = 0.002). There was a difference in sex distribution between those who reported SB (including suicidal ideation, plans, or attempts) and those who did not, with more females reporting SB compared to participants who did not (*P* < 0.001). There were no significant differences in residence or annual family income between participants with and without SB (*P* > 0.05).

**Table 1:** Demographic data and characteristics of the population with and without suicidal ideation.

	**Suicidal ideation**	**Non-suicidal ideation**	**T/χ**	***P***
	***N* = 3,002 (37.7%)**	***N* = 4,966 (62.3%)**		
Age (years)	16.09 ± 0.83	16.15 ± 0.83	3.138	0.002
Gender (*n*, %)			199.286	< 0.001
Male	1,207, 40.2%	2,807, 56.5%		
Female	1,795, 59.8%	2,159, 43.5%		
Residence (*n*, %)			3.623	0.058
Urban	526, 17.5%	789, 15.9%		
Rural	2,476, 82.5%	4,177, 84.1%		
Annual family income (*n*,%)			0.350	0.575
< 100,000 yuan	2,445, 81.4%	4,018, 80.9%		
More than 100,000 yuan	557, 18.6%	948, 19.1%		
Impulsivity	32.50 ± 10.69	25.59 ± 10.53	28.226	< 0.001
Insomnia (score)	8.13 ± 4.24	5.08 ± 3.34	35.676	< 0.001
Stress (score)	14.47 ± 9.39	7.74 ± 7.63	34.868	< 0.001
Anxiety (score)	13.08 ± 9.13	6.69 ± 7.03	35.056	< 0.001
Depression (score)	13.33 ± 9.64	6.31 ± 7.02	37.457	< 0.001
IA (score)	43.38 ± 13.22	35.62 ± 12.44	26.380	< 0.001

N/n, number of participants; IA, Internet addiction.

**Table 2:** Demographic data and characteristics of the population with and without a suicidal plan.

	**Suicidal plan**	**Non-suicidal plan**	**T/χ**	***P***
	***N* = 1,247 (15.7%)**	***N* = 6,721 (84.3%)**		
Age (years)	16.12 ± 0.83	16.13 ± 0.83	0.292	0.770
Gender (*n*, %)			28.255	< 0.001
Male	542, 43.5%	3,472, 51.7%		
Female	705, 56.5%	3,249, 48.3%		
Residence (*n*, %)			3.401	0.068
Urban	228, 18.3%	1,087, 16.2%		
Rural	1,019, 81.7%	5,634, 83.8%		
Annual family income (*n*, %)			0.015	0.937
< 100,000 yuan	1,013, 81.2%	5,450, 81.1%		
More than 100,000 yuan	234, 18.8%	1,271, 18.9%		
Impulsivity	32.80 ± 12.50	27.34 ± 10.62	16.217	< 0.001
Insomnia (score)	9.09 ± 5.11	5.70 ± 3.50	29.012	< 0.001
Stress (score)	15.84 ± 10.72	7.84 ± 7.34	34.868	< 0.001
Anxiety (score)	13.08 ± 9.13	6.69 ± 7.03	32.581	< 0.001
Depression (score)	16.31 ± 11.16	7.59 ± 7.53	37.457	< 0.001
IA (score)	43.91 ± 15.28	37.55 ± 12.63	15.77	< 0.001

N/n, number of participants; IA, Internet addiction.

**Table 3:** Demographic data and characteristics of the population with and without suicidal attempt.

	**Suicidal attempt** ***N* = 1,739 (21.8%)**	**Non-suicidal attempt** ***N* = 6,229 (78.2%)**	**T/χ**	***P***
Age (years)	16.07 ± 0.82	16.15 ± 0.83	3.061	0.002
Gender (*n*, %)			99.673	< 0.001
Male	692, 39.8%	3,322, 53.3%		
Female	1,047, 60.2%	2,907, 46.7%		
Residence (*n*, %)			0.769	0.381
Urban	299, 17.2%	1,016, 16.3%		
Rural	1,440, 82.8%	5,213, 83.7%		
Annual family income (*n*, %)			0.060	0.808
< 100,000 yuan	1,407, 80.9%	5,056, 81.2%		
More than 100,000 yuan	332, 19.1%	1,173, 18.8%		
Impulsivity	33.29 ± 11.72	26.77 ± 10.49	22.306	< 0.001
Insomnia (score)	8.90 ± 4.75	5.48 ± 3.40	33.720	< 0.001
Stress (score)	16.29 ± 10.11	8.60 ± 7.81	33.882	< 0.001
Anxiety (score)	15.03 ± 10.04	7.44 ± 7.16	35.535	< 0.001
Depression (score)	15.43 ± 10.55	7.15 ± 7.27	37.705	< 0.001
IA (score)	44.16 ± 14.30	36.97 ± 12.54	20.476	< 0.001

N/n, number of participants; IA, Internet addiction.

### 3.2. Spearman correlation analysis

Spearman correlation analysis showed that there were significant positive correlations between impulsivity, insomnia, DAS, Sym-T, Sym-C and Sym-W, RP-IH, RP-TM, and SB (including suicidal ideation, plans, or attempts; all *P* < 0.01; Table 4).

**Table 4:** Correlations between IA, impulsivity, stress, anxiety, depression, insomnia and SB.

	**Suicidal** **ideation**	**Suicide** **plan**	**Suicide** **attempt**
Impulsivity	0.308^**^	0.235^**^	0.163^**^
Stress	0.377^**^	0.336^**^	0.283^**^
Anxiety	0.389^**^	0.345^**^	0.294^**^
Depression	0.408^**^	0.361^**^	0.311^**^
Insomnia	0.381^**^	0.328^**^	0.269^**^
Sym-T	0.269^**^	0.142^**^	0.194^**^
Sym-C and Sym-W	0.272^**^	0.153^**^	0.208^**^
RP-IH	0.245^**^	0.123^**^	0.175^**^
RP-TM	0.276^**^	0.154^**^	0.211^**^

Sym-T, Internet addiction tolerance symptoms; Sym-C and Sym-W, compulsive Internet use and Internet addiction withdrawal symptoms; RP-IH, interpersonal and health problems; RP-TM, time management problems.

^**^ p < 0.01.

### 3.3. Structural equation modeling

**Figure 1:**
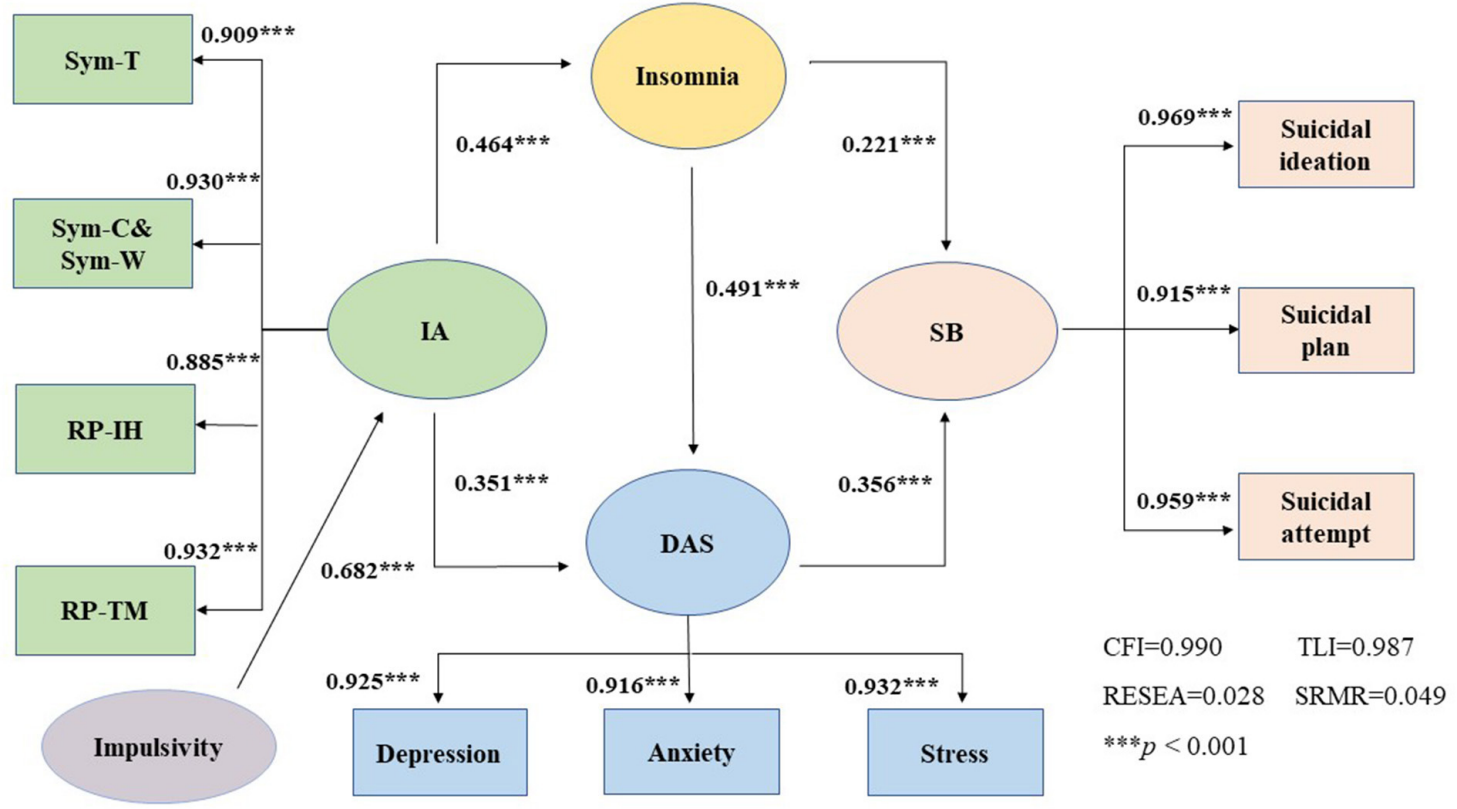
The SEM for the association between Internet addiction and suicidal behavior with insomnia, depression, anxiety, stress as mediating variables among Chinese vocational high school students (adjusted for age and sex as covariates). Sym-T, Internet addiction tolerance symptoms; Sym-C and Sym-W, compulsive Internet use and Internet addiction withdrawal symptoms; RP-IH, interpersonal and health problems; RP-TM, time management problems. IA, Internet addiction; DAS, depression, anxiety, and stress; SB, suicidal behavior. ***p < 0.001.

The path model showed that the standardized total effect of IA on the risk of SB was 0.309 (*P* < 0.001), with an indirect effect of 0.103 (*P* < 0.001) in the insomnia pathway, 0.125 (*P* < 0.001) in the DAS pathway, and 0.081 (*P* < 0.001) in insomnia → DAS pathway (Table 5). The proposed mediation model showed acceptable goodness of fit (CFI = 0.990, TLI = 0.987, RMSEA = 0.028, SRMR = 0.049). The SEM suggested that insomnia and DAS mediated the association between IA and SB. The final SEM image is shown in Figure 1. The SEM of the association between the IA factors (Sym-T, Sym-C & Sym-W, RP-IH, RP-TM) and SB is shown in the Supplementary material.

**Table 5:** Indirect effect of each mediate pathway from IA to suicidal behavior.

**Path**	**UC (SE)**	**SC (SE)**	***p-*Value**	**Effect size**
Total indirect effects	0.109 (0.003)	0.309 (0.009)	< 0.001	100%
IA → insomnia → SB	0.036 (0.002)	0.103 (0.007)	< 0.001	33.3%
IA → DAS → SB	0.044 (0.002)	0.125 (0.006)	< 0.001	40.5%
IA → insomnia → DAS → SB	0.029 (0.001)	0.081 (0.004)	< 0.001	26.2%

Effect size was calculated using the ratio of the total mediating effect.

UC, unstandardized coefficients; SC, standardized coefficient; SE, standard error; IA, Internet addiction; DAS, depression, anxiety, and stress; SB, suicidal behavior.

## 4. Discussion

This study examined the lifetime prevalence of SB among vocational high school students in China. It was the first to explore the relationship between IA and SB in detail by dividing the IA scale into core symptoms and addiction-related problems. After controlling for age and sex as covariates, the SEM results confirmed that IA was indirectly associated with SB through the mediating role of insomnia and/or DAS, and that there was no direct association between them.

### 4.1. Prevalence and demographic characteristics of SB

In this study, the lifetime prevalence of suicidal ideation, plans, and attempts among Chinese vocational high school students was 37.7, 15.7, and 21.8%, respectively. This is significantly higher than the lifetime prevalence of suicide ideation, planning, and attempts of global adolescents (18, 9.9, and 6.0%, respectively); (38), and also higher than that of Chinese high school students in other reports [26.8 (39), 13.2, and 5.2%, respectively] (40). The main reason for this difference could be the difference in sample sources. This study included students who entered vocational school immediately after middle school graduation. Although this group did not experience high school students' academic pressure, they tended to have worse academic performance, were considered underachievers with lower socioeconomic status, and faced more social challenges (such as low employment rate and educational attainment) and long-term social prejudice (41). This study found that the prevalence of suicide attempts was higher than that of plans. We speculated that some attempts were not extensively prepared and planned but were rather spontaneous and impulsive. Previous studies found that although people who have not made a suicide plan have a lower rate of suicide attempts, they account for 15–64% of all suicide attempts (42). Moreover, repeated suicide attempts may indicate a more serious intention to harm oneself, and hence, the behavior may be partly attention-seeking (43).

Moreover, three types of SB were more common in females, which is consistent with previous studies (44–46). Although the prevalence of female suicidal ideation, planning, and attempts is higher, the suicide death rate of males is much higher (47), and the risk of suicide completion after an attempt is also higher in males (48). This may be because females are generally more likely to seek help from friends and professionals, and be more willing to talk about emotional problems (49). The higher death rate among males could be because of more lethal methods of suicide (46) and less inclination to seek help (50).

### 4.2. Relationship between impulsivity and IA

We found that impulsivity is a strong predictor of IA. It is thought to be an endophenotype in individuals who develop addictive behaviors (e.g., pathological gambling and substance use disorder) (51) and our study provides theoretical support for this conclusion. A similar study found that individuals with IA showed higher levels of characteristic impulsivity, comparable to what was observed in pathological gamblers and that the severity of addiction was positively correlated with the level of impulsivity (52). Imaging data showed that participants with network game overuse had abnormal glucose metabolism in different brain areas, suggesting that overuse of the Internet and other types of addiction behavior (including substance and non-substance addiction) may have the same neurobiological mechanisms (53).

### 4.3. Mediating effects of insomnia and DAS on IA and SB

This study also found that SB was significantly and positively correlated with insomnia, DAS, and IA. Therefore, we conducted an SEM to further explore the relationship between these mental health problems and SB (54, 55). The SEM detected that under the mediating roles of DAS and insomnia, IA had indirect relationships with SB.

The first indirect path suggests that DAS mediated the relationship between IA and SB. Depression is one of the most reported factors associated with IA (56), and it may be a result of IA (57). We speculate that RP-TM caused by IA deprives the patient of the right to participate in real life. More time online means social withdrawal in real life, increased risk of social anxiety disorder, more conflicts with parents and peers, and a decline in emotional regulation ability (58, 59). Moreover, previous studies found that individuals with IA experience higher perceived stress (60, 61). They subsequently experienced multiple setbacks, such as impaired interpersonal relationships and failed exams, which may cause stress in students with IA (62). Available evidence on the core symptoms of IA is scarce. The withdrawal symptoms of substance addiction are known to cause emotional problems and physical discomfort (63). Our study also confirmed that Sym-C and Sym-W could cause insomnia and emotional problems. Resisting Sym-C also forced adolescents to experience stress. Considering the path from DAS to SB, many previous studies have confirmed that depression and anxiety are closely associated with increased suicide risk (64, 65). Many psychophysiological and neurobiological studies support the stress-driven suicide model (66, 67). Specifically, genetic stress susceptibility interacts with abnormal cortisol stress axis function and the inflammatory system, which may be an important biological mechanism of stressors that leads to SB (68).

The second indirect path suggests that insomnia mediated the relationship between IA and SB. IA is functionally equivalent to all addictions. In this study, Sym-T, Sym-C, Sym-W, and RP-TM were all found to be correlated with insomnia. A previous study suggested that when an individual cannot control the urge to use the Internet and bedtime is usually delayed, it often causes severe sleep deprivation or daytime sleepiness (69). Staying online in the middle of the night can lead to disturbed sleep-wake patterns (70). A study found that playing online games was associated with prolonged sleep latency and rapid eye movement sleep periods (69). Other potential mechanisms that disrupt sleep include bedtime Internet use, which may also affect the central and autonomic nervous systems (71), and screen light may inhibit the increase in melatonin, which promotes sleep (72). Moreover, surfing the Internet for a long time may cause many physical discomforts, such as dizziness, headache, and muscle pain, which may also affect sleep quality (73). The relationship between insomnia and suicide risk is unique, and studies suggest that insomnia may indirectly influence SB through specific biology (e.g., serotonergic dysfunction), psychology (mood disorders), cognitive deficits, and impulsivity (74).

Considering that insomnia can lead to mood disorders, the third mediating effect of “IA → insomnia → DAS → SB” can be explained as follows. In insomnia, the sleep-wake system (i.e., the circadian rhythm and homeostatic system) becomes dysfunctional, which may lead to abnormal mood regulation. Loss of sleep may increase the risk of SB by affecting the regulation of emotional responses (75). For example, a study supporting this conclusion reported that the mean incubation period of rapid eye movement was significantly shorter and its percentage was significantly higher in depression patients with suicidality than in patients without, which also indicates that suicidal patients may not be able to self-regulate their emotions during sleep (76). However, the mediating mechanism of emotional problems in the relationship between insomnia and SB requires further study.

This study has some limitations. First, it only recruited students from vocational schools in Hunan Province; therefore, the sample may not represent the entire population of vocational students in China. Second, self-report scales were used in this study, and clinical diagnosis required professional interviews. Third, since this study had a cross-sectional design, besides, the relationship between IA and SB was unclear, the causal relationship between the variables could not be determined. Future longitudinal studies are needed to explore the causal relationship and influencing factors between IA and SB. Fourth, we evaluated lifelong suicidal behavior and in future studies, we will assess suicidal behavior in the past 6 months and the past year does better illustrate the relationship between Internet addiction and suicidal behavior.

In conclusion, this study explored the relationship between IA, insomnia, DAS, and SB using a mediation model, and provided some evidence for the process of IA leading to SB. This study also provides a basis for the prevention of SB in individuals with IA and can help reduce the risk of suicide in individuals with IA by improving sleep quality, enhancing their ability to deal with stress, and alleviating emotional problems.

## Data availability statement

The raw data supporting the conclusions of this article will be made available by the authors, without undue reservation.

## Ethics statement

The studies involving human participants were reviewed and approved by the Ethics Committee of Second Xiangya Hospital, Central South University, China. Written informed consent to participate in this study was provided by the participants' legal guardian/next of kin.

## Author contributions

ZT, JH, and JC had the original idea for the study and designed the survey. YZ wrote the manuscript. ZW and RW revised the survey and the design. MG, MT, YZ, ML, JK, ZC, YY, RL, and ZC were involved in the data collection. JH is the principal investigator of this clinical trial. All authors read and approved the final version of the manuscript.
