# Supplementary material for: Internet addiction and suicidal behavior among vocational high school students in Hunan Province, China: A moderated mediation model
Source: Front Public Health. 2023 Jan 10;10:1063605. doi: 10.3389/fpubh.2022.1063605 (PMC9871611; doi:10.3389/fpubh.2022.1063605)
Supplement: Supplementary file 1 [file Data_Sheet_1.PDF]

Supplementary

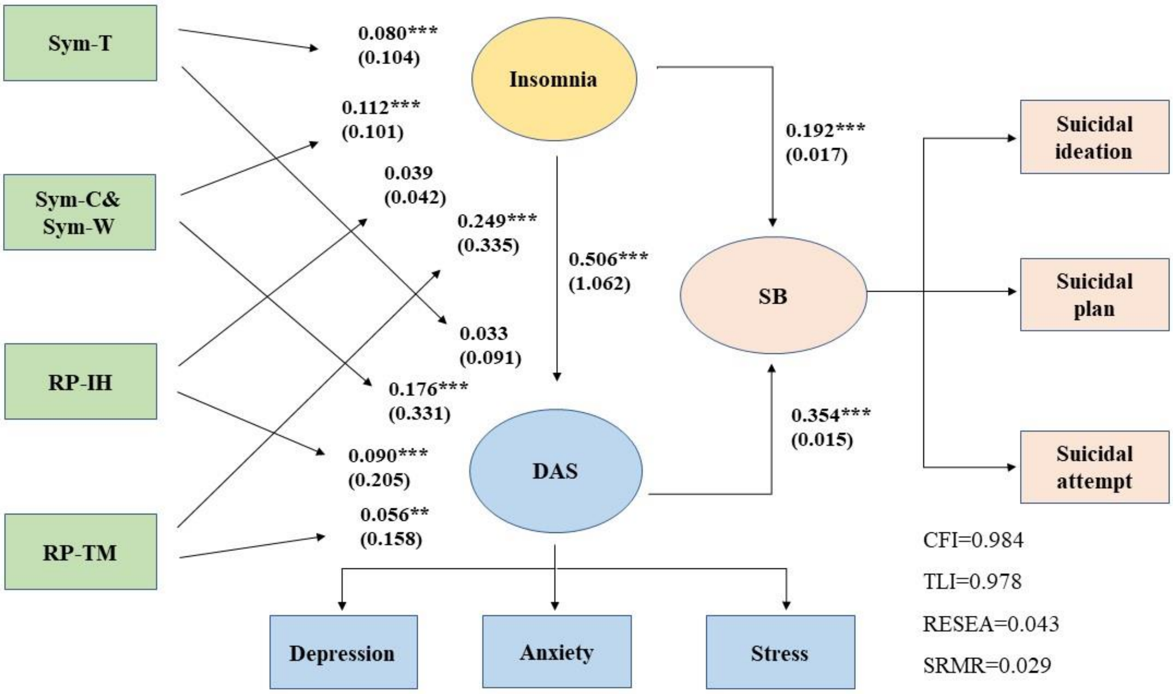

The SEM for the association between Internet addiction factors (Sym-T, Sym-C & Sym-W, RP-IH, RP-TM) and suicidal behavior with insomnia, depression, anxiety, stress as mediating variables among Chinese vocational high school students (adjusted for age and sex as covariables).

Abbreviations: Sym-T, Internet addiction tolerance symptoms; Sym-C and Sym-W, compulsive Internet use and Internet addiction withdrawal symptoms; RP-IH, interpersonal and health problems; RP-TM, time management problems. DAS, depression, anxiety, and stress; SB, suicidal behavior.

***p*<0.01, *** *p* < 0.001
